# Supplementary material for: Saxifraga spinulosa-Derived Components Rapidly Inactivate Multiple Viruses Including SARS-CoV-2
Source: Viruses. 2020 Jun 28;12(7):699. doi: 10.3390/v12070699 (PMC7411974; doi:10.3390/v12070699)
Supplement: Supplementary file 1 [file viruses-12-00699-s001.pdf]

Table S1 The sequence of primers used in this study and each PCR condition.

Primers name	Primer sequences	PCR condition
IAV-Primer set 1<br>(982 bp)	Fwd: 5′-ATGAGTCTTCTAACCGAGGTC-3′<br>Rev: 5′-GTCAGCATAGAGCTGGAGTAA-3′	95°C for 5 min<br>↓<br>[95°C for 30 sec, 52°C for 30 sec, 72°C for 1 min] x 25, 24 times (Figure 2A, 2C)<br>↓<br>72 °C for 10 min
IAV-Primer set 2<br>(253 bp)	Fwd: 5′-AAGACCAATCCTGTCACCTC-3′<br>Rev: 5′-CAGTTGTATGGGCCTCATATAC-3′	95°C for 5 min<br>↓<br>[95°C for 30 sec, 52°C for 30 sec, 72°C for 1 min] x 22 times<br>↓<br>72 °C for 10 min
IAV-Primer set 3<br>(320 bp)	Fwd: 5′-ACAGATTGCTGACTCCCA-3′<br>Rev: 5′-TGATCCTCTCGCTATTGCC-3′	95°C for 5 min<br>↓<br>[95°C for 30 sec, 52°C for 30 sec, 72°C for 1 min] x 22 times<br>↓<br>72 °C for 10 min
FCV-Primer set<br>(264 bp)	Fwd: 5′-TCCACACTAGCGTCAACTGG-3′<br>Rev: 5′-GACGAGCGTCAAACAGAACA-3′	95°C for 5 min<br>↓<br>[95°C for 30 sec, 49°C for 30 sec, 72°C for 1 min] x 32 times<br>↓<br>72 °C for 10 min
MNV-F1 and -R1<br>[1] (721 bp)	Fwd: 5′-GCCATGCATGGTGAAAAG-3′<br>Rev: 5′-CATGCARACCAGGCGCATAG-3′	95°C for 5 min<br>↓<br>[95°C for 30 sec, 49°C for 30 sec, 72°C for 1 min] x 33 times<br>↓

		72 °C for 10 min
NIID_2019-nCoV _N_F2 and R2 (158 bp)	Fwd: 5′-AAATTTTGGGGACCAGGAAC-3′<br>Rev: 5′-TGGCAGCTGTGTAGGTCAAC-3′	95°C for 5 min<br>↓<br>[95°C for 30 sec, 49°C for 30 sec, 72°C for 1 min] x 30 times<br>↓<br>72 °C for 10 min

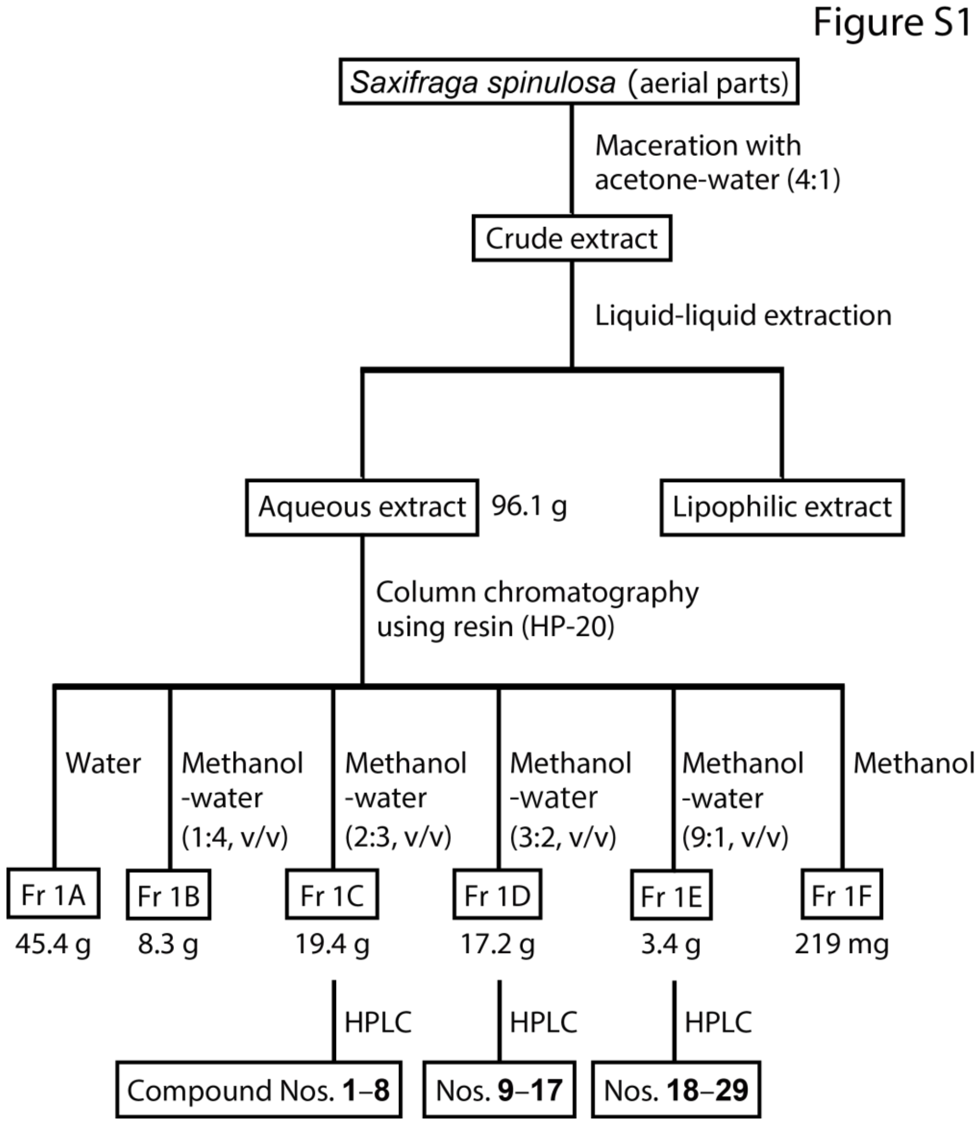

Figure S1. Scheme of the sample extraction and isolation.

Figure S2

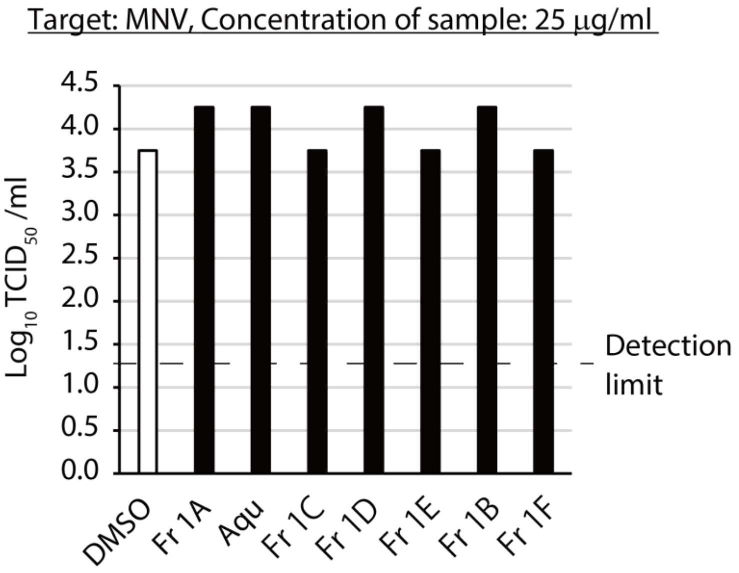

Figure S2. Evaluation of the MNV-inactivating activity of 25 μg/ml *SS*-derived fractions. The *SS*-derived fractions and DMSO control were added to solutions containing MNV followed by incubation at 25°C for 48 h. Viral titer was then evaluated. Aqu: Aqueous extract.

A

Figure S3

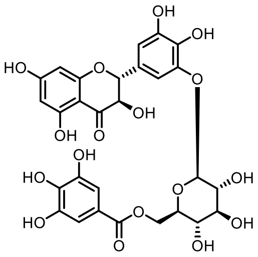

Compound **1**

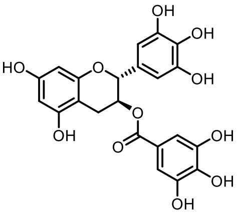

Compound **2**

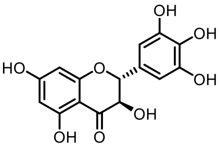

Compound **3**

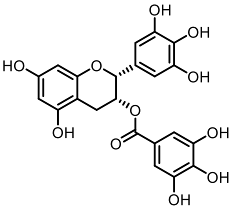

Compound **4**

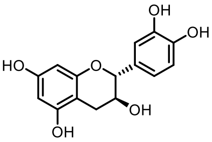

Compound **5**

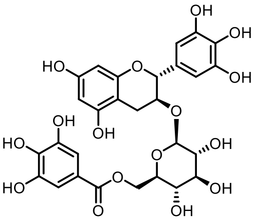

Compound **6**

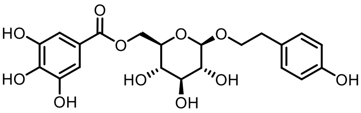

Compound **7**

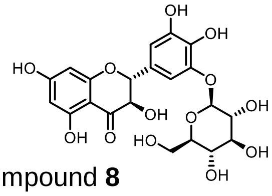

Compound **8**

B **Figure S3**

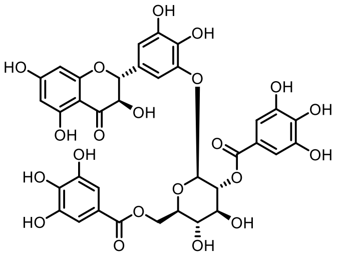

Compound **9**

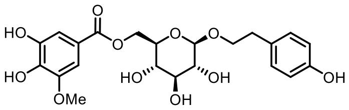

Compound **10**

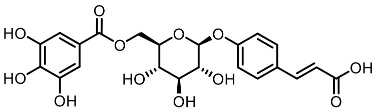

Compound **11**

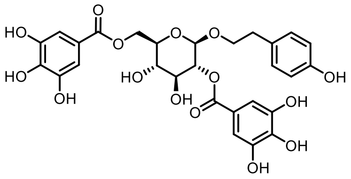

Compound **12**

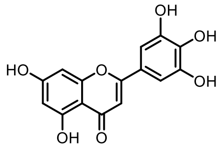

Compound **13**

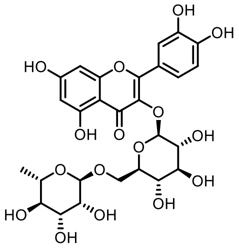

Compound **14**

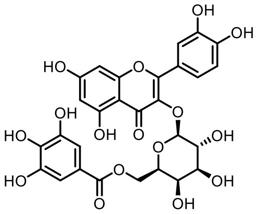

Compound **15**

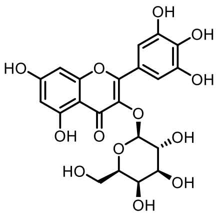

Compound **16**

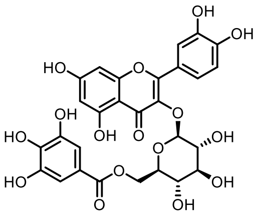

Compound **17**

C **Figure S3**

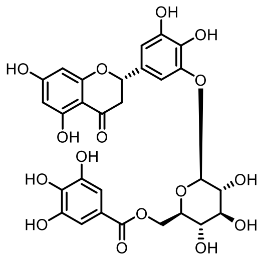

Compound **18**

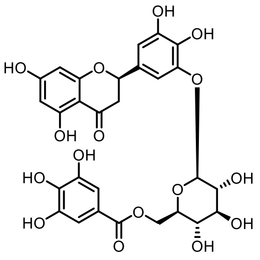

Compound **19**

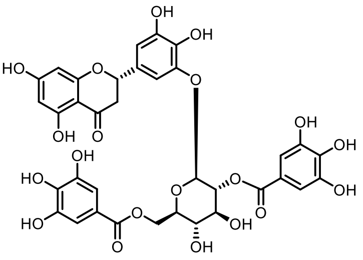

Compound **20**

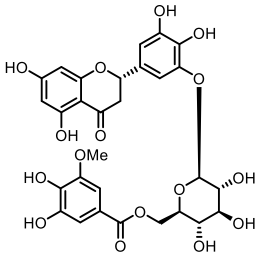

Compound **21**

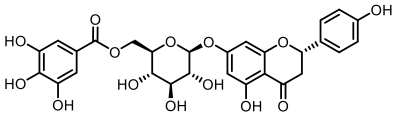

Compound **22**

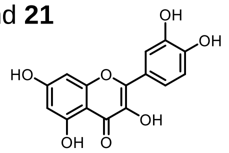

Compound **23**

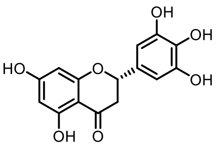

Compound **24**

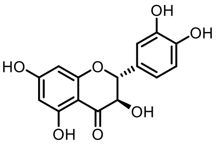

Compound **25**

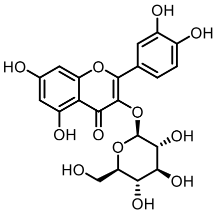

Compound **26**

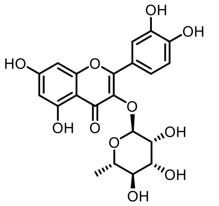

Compound **27**

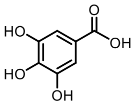

Compound **28**

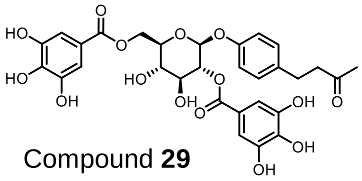

Compound **29**

Figure S3. Chemical structures of compounds isolated from *SS*.
(**A**–**C**) Compounds isolated from (**A**) Fr 1C, (**B**) Fr 1D, and (**C**) Fr 1E.

Figure S4

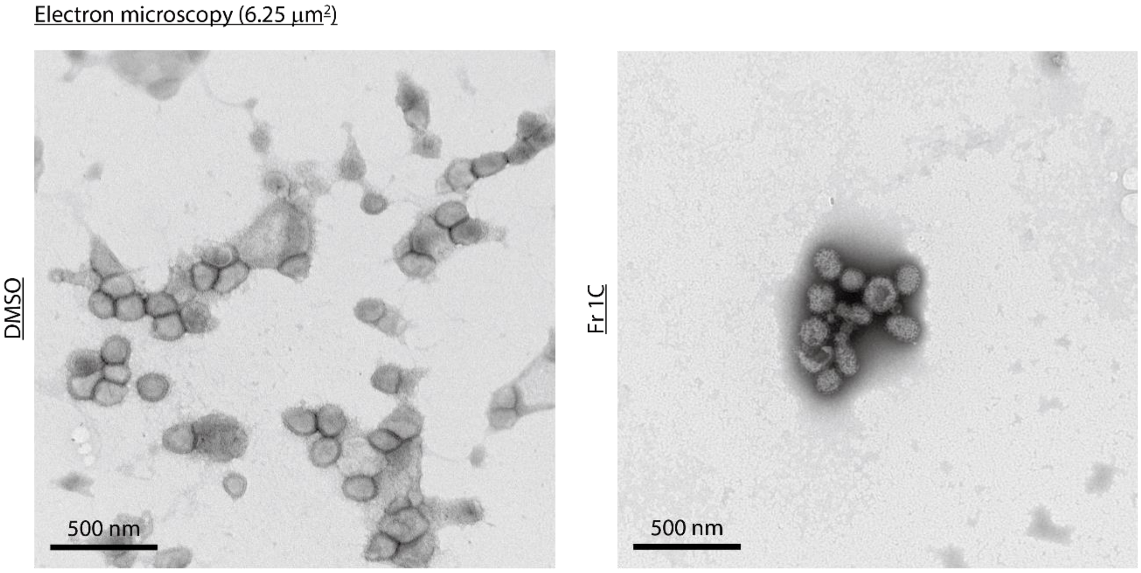

Figure S4. Electron microscopic images of IAV under low magnification (see Figure 3). The IAV virions featured in Figure 3 were evaluated using transmission electron microscopy under low magnification. The panels to the left and right include representative images of DMSO- and Fr 1C-treated virion particles, respectively in 6.25 μm^2 fields.

Figure S5

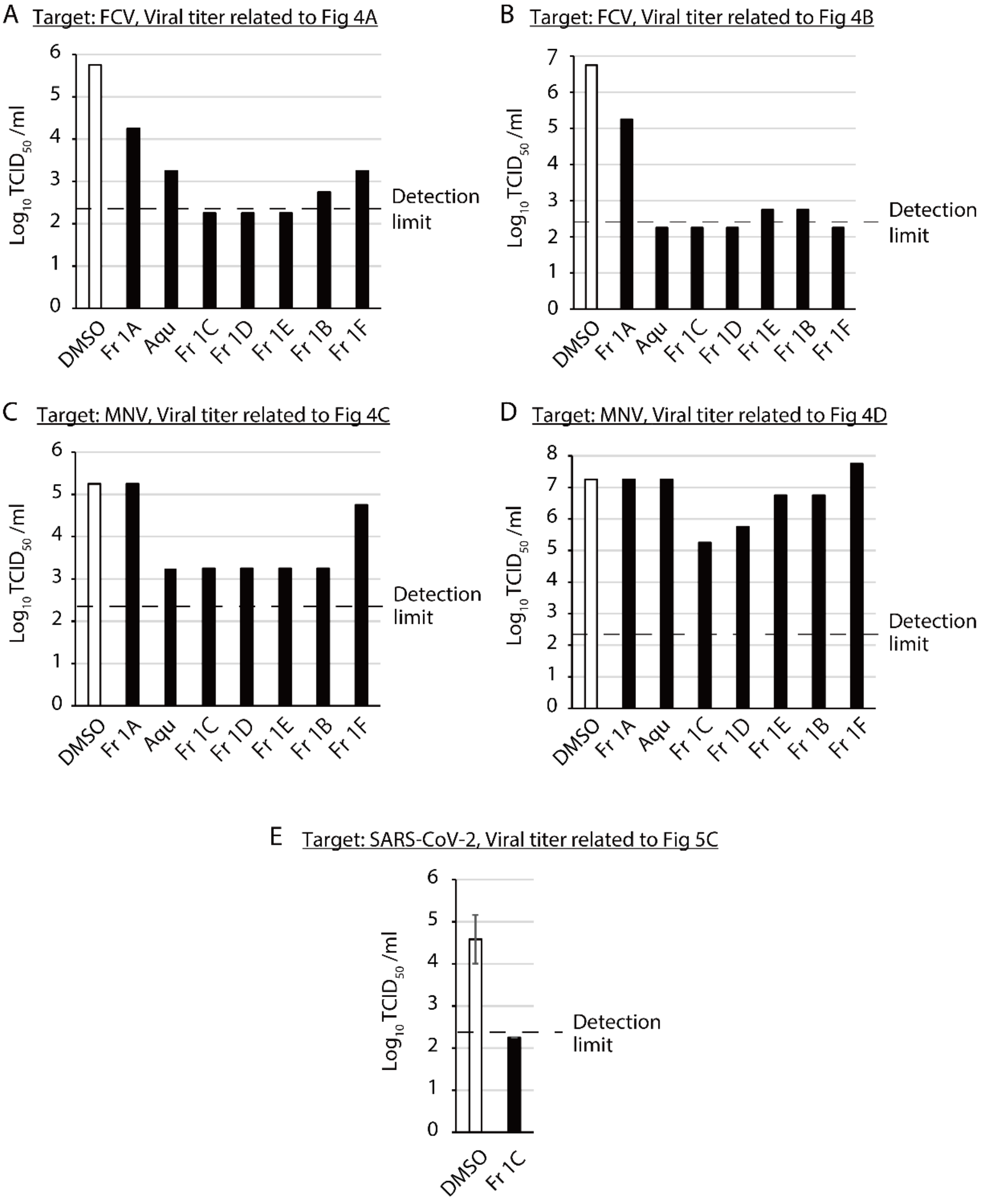

Figure S5. The viral titers of FCV, MNV, and SARS-CoV-2 treated with *SS*-derived fractions (see Figures 4 and 5).
(**A**–**E**) Viral titer of the viral mixtures in (**A**) Figure 4A, (**B**) Figure 4B, (**C**) Figure 4C, (**D**) Figure 4D, and (**E**) Figure 5C. Aqu: Aqueous extract.

Reference

1. Kitajima, M.; Oka, T.; Tohya, Y.; Katayama, H.; Takeda, N.; Katayama, K. Development of a broadly reactive nested reverse transcription-PCR assay to detect murine noroviruses, and investigation of the prevalence of murine noroviruses in laboratory mice in Japan. *Microbiol. Immunol.* **2009**, 53, 531-534, doi:10.1111/j.1348-0421.2009.00152.x.
